# Opioid metabolism and drug-drug interaction in cancer

**DOI:** 10.1093/oncolo/oyae094

**Published:** 2024-05-23

**Authors:** Matti Aapro, Stefano Fogli, Bart Morlion, Romano Danesi

**Affiliations:** Genolier Cancer Centre, Clinique de Genolier, 1272 Genolier, Switzerland; Department of Clinical and Experimental Medicine, University of Pisa, 56126 Pisa PI, Italy; Department of Cardiovascular Sciences, Section Anesthesiology and Algology, University of Leuven, 3000 Leuven, Belgium; Department of Oncology and Hemato-Oncology, University of Milano, 20122 Milano MI, Italy

**Keywords:** opioid, analgesics, pain, drug-drug interactions, anticancer drugs, drug interaction checker

## Abstract

Concomitant use of multiple drugs in most patients with cancer may result in drug-drug interactions (DDIs), potentially causing serious adverse effects. These patients often experience unrelieved cancer-related pain (CRP) during and after cancer treatment, which can lead to a reduced quality of life. Opioids can be used as part of a multimodal pain management strategy when non-opioid analgesics are not providing adequate pain relief, not tolerated, or are contraindicated. However, due to their narrow therapeutic window, opioids are more susceptible to adverse events when a DDI occurs. Clinically relevant DDIs with opioids are usually pharmacokinetic, mainly occurring via metabolism by cytochrome *P450* (CYP). This article aims to provide an overview of potential DDIs with opioids often used in the treatment of moderate-to-severe CRP and commonly used anticancer drugs such as chemotherapeutics, tyrosine kinase inhibitors (TKIs), or biologics. A DDI-checker tool was used to contextualize the tool-informed DDI assessment outcomes with clinical implications and practice. The findings were compared to observations from a literature search conducted in Embase and PubMed to identify clinical evidence for these potential DDIs. The limited results mainly included case studies and retrospective reviews. Some potential DDIs on the DDI-checker were aligned with literature findings, while others were contradictory. In conclusion, while DDI-checkers are useful tools in identifying potential DDIs, it is necessary to incorporate literature verification and comprehensive clinical assessment of the patient before implementing tool-informed decisions in clinical practice.

## Teaser

Considering the common prescription of multiple medications in patients with cancer-related pain, it is important for healthcare providers to understand the risk of potential drug-drug interactions (DDIs) between opioids and concomitantly used anticancer agents. This review analyzes the outcome of tool-informed assessment of DDIs compared to evidence from the literature.

**Implications for practice**

Considering the common prescription of multiple medications in patients with cancer-related pain, it is important for healthcare providers (HCPs) to understand the risk of potential drug-drug interactions (DDIs) between opioids and concomitantly used anticancer agents. The clinical consequences of such DDIs may include toxicity or a decreased analgesic effect, which have important implications for HCPs when making treatment decisions. This review analyzes the outcome of tool-informed assessment of DDIs compared to evidence from the literature, to elucidate gaps and provide potential solutions and guidance to HCPs in clinical practice.

## Introduction

Drug-drug interactions (DDIs) are common in patients with cancer because of multiple drugs they take concomitantly.^1-3^ Polypharmacy (concomitant use of 5 drugs or more) increases the risk for DDIs and severe adverse effects.^4-6^ A European cross-sectional study reported that 84.4% (N = 1923) of patients with cancer receive 5 or more concomitant drugs.^7^ These drugs include cytotoxic, targeted, hormonal, and supportive care agents in addition to the drugs prescribed to treat multimorbidity.^2^

Despite advances in early diagnosis and treatment of cancer, cancer-related pain (CRP) commonly occurs, which can lead to reduced quality of life.^8-11^ Multimodal pain management can include opioids, particularly recommended for the treatment of moderate-to-severe CRP when non-opioid analgesics are not providing adequate pain relief, not tolerated, or are contraindicated.^12-14^ Opioids have a narrow therapeutic index, which makes them more susceptible to adverse events when a DDI occurs.^15,16^ DDIs with opioids can lead to an exacerbation of adverse effects or reduced analgesic efficacy.^17,18^ Pharmacokinetic (PK) DDIs, occurring when one drug impacts the absorption, distribution, metabolism, or excretion of another, represent the majority of clinically relevant DDIs with opioids, while pharmacodynamic (PD) DDIs are rarely of clinical relevance.^2,15,19^

PK-based DDIs primarily occur through altered opioid phase I metabolism by cytochrome P450 (CYP), and often lead to changes in plasma concentrations.^15^ A CYP inhibitor administered with a prodrug opioid would lead to decreased metabolism to its active form and potential treatment failure, whereas concentration-dependent toxicity may occur when the opioid is an active parent drug.^15^ Other PK DDIs are due to altered phase II metabolism (eg, glucuronidation) or drug absorption regulated by the efflux pump transmembrane protein P glycoprotein (P-gp).^15,19^

Inhibitors and inducers of the latter can promote or reduce the absorption of some opioids.^20^

Therefore, it is crucial for healthcare providers (HCPs) to understand the risk of potential DDIs and clinical consequences that can occur between opioids and concomitantly used drugs.^18^

This article aims to provide an overview of potential DDIs with opioids often used in the treatment of moderate-to-severe CRP and other commonly used anticancer drugs, such as chemotherapeutics, tyrosine kinase inhibitors (TKIs), or biologics. A DDI-checker tool was used to put the outcome of tool-informed assessments of DDIs into context with clinical implications and practice.

## Materials and Methods

An open access interaction checker website (Cancer—Drug interactions checker, https://cancer-druginteractions.org/checker),^21^ a tool designed by Radboud University Medical Center and University of Liverpool to check for interactions with anticancer drugs (in the following referred to as DDI-checker), was used to initially identify potential DDIs between opioids and selected anticancer drugs. The DDI-checker was selected for this study due to accessibility, and the system used to evaluate the quality of evidence for potential DDIs was the Grading of Recommendations, Assessment, Development, and Evaluation (GRADE) (Table S1). According to the website, the information provided by the tool is based on thorough review of relevant published and unpublished data, including controlled clinical DDI studies, probe substrate studies, as well as Summaries of Product Characteristics (SmPC) and US Prescribing Information (USPI) licenses.^21^ The DDI-checker was last updated in June 2022.

On the DDI-checker, the impact of cancer drugs on opioids was evaluated to determine the risks of opioid toxicity or lack of efficacy. Interactions were classified for specific drug combinations using the “traffic light” analogy according to likelihood and severity, ie, should not be coadministered (red), potential clinically significant interaction (amber), potential interaction likely but of weak intensity (yellow), and no clinically significant interaction expected (green).^21^ Figures were created for pairs of opioids and anticancer drugs with red and amber DDIs, considered clinically significant. Management advice on the potential clinically significant DDIs was provided where needed.^21^

A literature search was conducted in Embase and PubMed from January 1990 until March 2023 to identify clinical evidence for DDIs expected for each pair of opioid and anticancer drugs (refer to Supplementary Data 2 for the full search strategy; Supplementary Tables S2 and S3). Randomized controlled trials (RCTs), observational studies, retrospective studies, case reports, abstracts, and congress data were included. Search terms included frequently used opioids for moderate-to-severe CRP,^13^ and selected anticancer drugs for treatment of common types of cancer such as female breast, lung, and prostate cancers.^22,23^

Opioids included buprenorphine, fentanyl, hydrocodone, methadone, morphine, oxycodone, and tramadol. Anticancer drugs included chemotherapeutics (taxanes, specifically paclitaxel and docetaxel; platinum agents, specifically oxaliplatin, carboplatin, and cisplatin; oxazophosphorines, specifically cyclophosphamide; and vinca alkaloids, specifically vincristine), TKIs (dasatinib, gefitinib, imatinib, and nilotinib), biologics (atezolizumab, nivolumab, and trastuzumab), cyclin-dependent kinase 4/6 inhibitors (palbociclib and ribociclib), estrogen receptor modulators (tamoxifen), steroid hormones (abiraterone), and nonsteroidal antiandrogens (enzalutamide).

DDI terms were drug interaction(s), drug-drug interaction(s), drug antagonism, polypharmacy, drug synergism(s), drug potentiation(s), drug augmentation(s), drug toxicity, drug competition, drug inhibition, and drug intoxication.

The findings on the DDI-checker were manually compared to observations from the literature search to identify clinical evidence for potential DDIs.

The authors’ expert views on the use of internet checker tools and databases in the assessment of DDIs and guiding treatment decisions in clinical practice were compiled and summarized in the *Expert Opinion* section.

## Results

Most of the identified opioid DDIs from the DDI-checker are related to their CYP metabolism, mainly involving CYP3A4 and CYP2D6, and few are related to glucuronidation by uridine diphosphate glucuronosyltransferase (UGT).^21^

### Buprenorphine

Buprenorphine is metabolized through *N*-dealkylation via CYP3A4 to form the active metabolite norbuprenorphine. Both buprenorphine and norbuprenorphine are further modified through glucuronidation by UGT2B7 and UGT1A1, respectively.^21,24^ Buprenorphine is a partial agonist at the μ opioid receptor and an antagonist at the κ opioid receptor. Like other μ agonists, it may cause respiratory depression, miosis, and mood changes, in addition to analgesia. However, regarding respiratory outcome, there appears to be a ceiling effect with high doses.^25,26^ Norbuprenorphine is a potent agonist of μ, δ, and κ opioid receptors and, in animal models, may cause greater respiratory depression while having reduced antinociception, compared with buprenorphine.^27^

**Figure 1.**
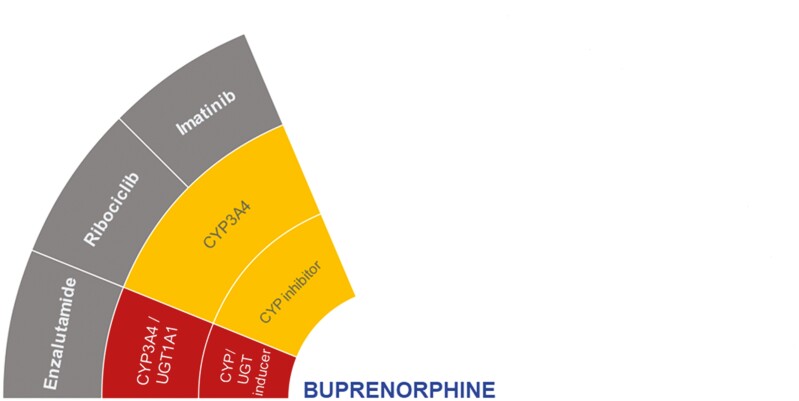
Proposed DDIs between buprenorphine (center) and selected anticancer drugs (gray) categorized as “should not be coadministered” (red) or ‘potentially clinically significant’ (amber) according to the DDI-checker. The inner rings indicate relevant enzymes and transporters involved and whether anticancer drugs act as inhibitors or inducers. Abbreviations: CYP, cytochrome *P450*; UGT, uridine diphosphate glucuronosyltransferase.

According to the DDI-checker, buprenorphine DDIs that have not been studied but may be clinically significant involve coadministration with enzalutamide, imatinib, and ribociclib (Figure 1).^21^ Enzalutamide is a strong CYP3A4 inducer and can also induce UGT1A1.^21^ This may increase the formation of active buprenorphine metabolites leading to toxicity.^21^ Imatinib and ribociclib (both CYP3A4 inhibitors) may increase concentrations of buprenorphine and decrease norbuprenorphine. Coadministration, if unavoidable, should be closely monitored for buprenorphine toxicity.^21^

The level of CYP3A4 inhibition is dependent on the dose of ribociclib administration (eg, strong at 600 mg and moderate at 400 mg).^28,29^ There is a sparsity of data to support findings on the DDI-checker.

### Fentanyl

**Figure 2.**
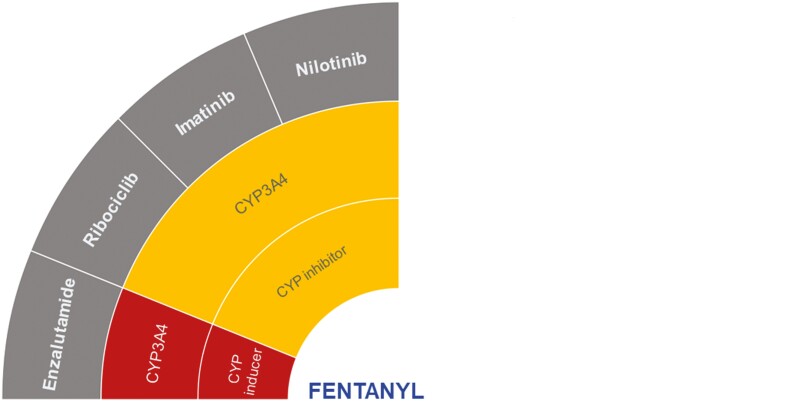
Proposed DDIs between fentanyl (center) and selected anticancer drugs (gray) categorized as “should not be coadministered” (red) or ‘potentially clinically significant’ (amber) according to the DDI-checker. The inner rings indicate relevant enzymes and transporters involved and whether anticancer drugs act as inhibitors or inducers. Abbreviation: CYP, cytochrome *P450*.

Fentanyl is an opioid receptor agonist, mainly metabolized by CYP3A4 to its non-active metabolite, norfentanyl.^30,31^ Proposed DDIs according to the DDI-checker, which may be clinically significant were found with enzalutamide, nilotinib, ribociclib, and imatinib (Figure 2).^21^

As a strong CYP3A4 inducer, enzalutamide is contraindicated with fentanyl as it may lead to a significant decrease in fentanyl concentrations which is unlikely to be compensated by dose adjustment.^21^ This was supported by a small clinical study (*N* = 8) that found fentanyl concentrations undetectable when coadministered with enzalutamide versus abiraterone acetate. The latter served as a control since no DDI was expected (Table 1).^32^ In patients treated with enzalutamide, undetectable fentanyl concentrations resulted in loss of analgesic effect, and opioids like morphine that are not metabolized by CYP3A4 were suggested as alternatives.^32^ A case study has shown reduced fentanyl analgesia in a patient suffering from metastasized castration-resistant prostate cancer (CRPC) receiving enzalutamide (Table 1).^33^ The lack of analgesia persisted despite increasing the fentanyl transdermal dose, prompting discontinuation of enzalutamide treatment. Consequently, the cervical neuropathic pain became more manageable.^33^

**Table 1.** Clinical evidence from literature search findings highlighting potential DDIs between opioids and selected anticancer drugs.

Opioid	Clinical evidence			
	Author (year)	Type of study	Patient case/study population	Main findings
Buprenorphine	NA			
Fentanyl	Benoist (2019)^32^	Comparative, 2-arm parallel study	CRPC (N = 8) Arm 1: *fentanyl *+ *enzalutamide* (n = 6) Arm 2: *fentanyl* + *abiraterone acetate* (n = 2)	Plasma fentanyl concentrations measured
				Undetectable fentanyl concentrations in arm 2; potential loss of analgesic effect Coadministration of enzalutamide with fentanyl and other CYP3A4-metabolized opioids to be avoided
	Westdorp (2018)^33^	Case study	62-year old with CRPC and progressive bone and liver metastases *Enzalutamide* → hospitalisation due to severe pain from cervical spine bone metastases → dexamethasone (16 mg, OD), palliative radiotherapy (8 Gy, single dose), *fentanyl* (transdermal + oromucosal), pregabalin, + amitriptyline → increasing fentanyl dose due to non-responsiveness—*fentanyl* (transdermal, 75 mg, hourly)	Insufficient analgesia with increased dose of fentanyl Enzalutamide stopped 4 weeks after admission Patient discharged 8 weeks later when cervical neuropathic pain was manageable compared to the in-hospital setting Caution must be exercised during coadministration of enzalutamide and CYP3A4 substrates
Hydrocodone	NA			
Methadone	Ferretti (2004)^34^	Case study	50-year-old male with Chemotherapy resistant stage IV bronchioalveolar lung carcinoma; opioid addiction managed by methadone maintenance *Methadone:* > 30 years *Gefitinib:* prescribed 50 days before admission	Symptoms at admission: confusion, persistent hypotension; patient received oxygen, bronchodilators, dexamethasone, ranitidine, phenobarbital Overdose syndrome on fifth in-hospital day; methadone stopped, developed withdrawal symptoms, methadone reintroduced; patient displayed tachypnea then died of respiratory failure After initiating gefinitib, attention should be paid to clinical respiratory symptoms and radiographic findings on patients treated with methadone
Morphine	NA			
Oxycodone	Weme (2022)^35^	Prospective, 2-arm parallel study	Prostate cancer (N = 26) Arm 1: *oxycodone* (normal release, 15 mg) + *enzalutamide* (160 mg, OD, 40 days) (n = 13) Arm 2: *oxycodone* (normal release, 15 mg) (n = 13)	Plasma concentrations of oxycodone and its metabolites measured Significant decrease of oxycodone and oxymorphone concentrations in arm 1 compared to arm 2 patients Enzalutamide discontinuation may cause oxycodone overdose Switch from oxycodone to non-CYP3A4 metabolized opioid (eg, morphine) required for efficacious and safe pain management
	Westdorp (2018)^33^	Case study	CRPC 70-year-old; progressive bone disease *Enzalutamide* (160 mg, OD)→ fixed-dose paracetamol + oxycodone (immediate release, 5 mg if required)→ palliative radiotherapy (8 Gy, single dose) → *oxycodone* (controlled-release low-dose) → increasing dosage due to non-responsiveness—*oxycodone* (20 mg, BID) + escape medication → dexamethasone	Analgesic treatment was still insufficient Opioid rotation with morphine sulfate (30 mg, BID) = total pain relief within 24 hours with no escape medication For non-responsiveness from opioid + enzalutamide, opioid rotation must be considered in favor of non-CYP3A4-metabolized opioids (eg, morphine)
	Westdorp (2018)^33^	Case study	CRPC 72-year-old; progressive bone disease Bicalutamide and nilutamide→ *enzalutamide* (160 mg, OD)→ painful bone metastases on the spine + pelvis (after 4 month) → paracetamol, diclofenac, and oxycodone (controlled-release) + oxycodone (immediate-release)	Analgesic treatment was insufficient Opioid rotation with morphine sulfate (low-dose, 10 mg, BID); pain relief in 48 hours For non-responsiveness from opioid + enzalutamide, opioid rotation must be considered in favor of non-CYP3A4-metabolized opioids (eg, morphine)
	Lee (2015)^36^	Retrospective review	CRPC (N = 69) *enzalutamide* + *oxycodone* (n = 5) 2 databases, Lexicomp and Micromedex, were used to analyze DDIs from individual drug histories from pharmacy records	Moderate risk of DDI with enzalutamide + oxycodone identified by Micromedex Lexicomp listed enzalutamide + oxycodone as potential DDI of clinical importance Further studies required for conclusive evidence of clinically significant DDI
	Jamani (2015)^37^	Retrospective review	CRPC (N = 91) *Abiraterone acetate* + *oxycodone* (n = 4) 2 databases, Lexicomp and Micromedex, were used to analyze DDIs from individual drug histories from pharmacy records	Major risk of DDI with abiraterone and oxycodone identified by Micromedex Among most flagged clinically significant potential DDIs according to Lexicomp More studies with larger populations needed to establish clinical significance of the DDI
Tramadol	Bodega-Azuara (2022)^38^	Retrospective review	Prostate cancer (N = 32) with antiandrogenic treatment: *abiraterone* (n = 21), apalutamide (n = 3) and *enzalutamide* (n = 8) Use of Liverpool and Uptodate (Lexicomp) databases to evaluate DDIs involving cardiovascular drugs, antithrombotics, proton pump inhibitors, analgesics	Anticancer drugs with clinically relevant DDIs with opioids and other drugs n = 18 *Abiraterone* (11.1%) Apalutamide (33.3%%) *Enzalutamide* (55.6%) Abiraterone/apalutamide to be administered with analgesic other than tramadol due to safety or efficacy concerns

Abbreviations: BID, twice daily; CML, chronic myelogenous leukemia; CRPC, castration-resistant prostate cancer; CYP, cytochrome *P450*; DDI, drug-drug interaction; Gy, gray; NA, not available; OD, once daily.

Fentanyl DDIs with nilotinib, imatinib, and ribociclib (all amber) (Figure 2),^21^ lack supporting evidence from clinical studies. Nilotinib, imatinib, and ribociclib are moderate CYP3A4 inhibitors and may cause potential increase in fentanyl concentrations. This may result in potential fentanyl-related toxicity.^21^ However, DDIs analyzed in healthy participants showed that 600 mg ribociclib is a strong CYP3A4 inhibitor.^39^

### Hydrocodone

**Figure 3.**
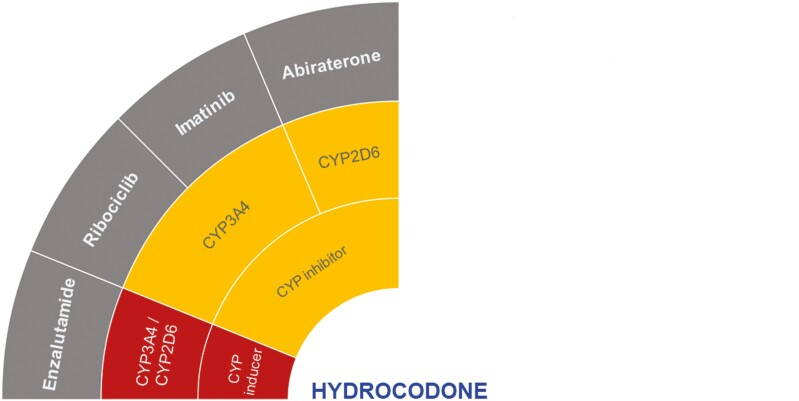
Proposed DDIs between hydrocodone (center) and selected anticancer drugs (gray) categorized as “should not be coadministered” (red) or ‘potentially clinically significant’ (amber) according to the DDI-checker. The inner rings indicate relevant enzymes and transporters involved and whether anticancer drugs act as inhibitors or inducers. Abbreviation: CYP, cytochrome *P450*.

Hydrocodone is mainly metabolized by CYP2D6 to its active metabolite, hydromorphone, via *O*-demethylation.^40,41^ The hydrocodone metabolism and therapeutic efficacy may be influenced by the genetic polymorphisms of CYP2D6.^41^ Another active metabolite, norhydrocodone, is formed via *N*-demethylation by CYP3A4.^41,42^ Hydromorphone is further modified through glucuronidation to hydromorphone-3-glucuronide (main metabolite).^40^ Although coadministration with the selected anticancer drugs has not been studied, hydrocodone, but not hydromorphone, may potentially have clinically significant DDIs with enzalutamide, imatinib, abiraterone, and ribociclib according to the DDI-checker (Figure 3).^21^

As a strong and moderate inducer of CYP3A4 and CYP2D6, respectively, enzalutamide may lead to supratherapeutic exposure by increasing hydromorphone and norhydrocodone levels. This potential DDI may cause toxicity.^21^ Both imatinib and ribociclib (dose-dependent) are likely to increase hydrocodone concentrations through CYP3A4 inhibition. Abiraterone would have a similar effect through CYP2D6 inhibition. If coadministration is unavoidable, patients must be closely monitored for toxicity.^21^ For abiraterone in particular, a 50% dose reduction of hydrocodone is suggested according to the DDI-checker.^21^ However, it is worth noting that this is an example of inaccurate reporting of decrease/increase of prodrug versus parent drug as a consequence of a potential DDI according to the DDI-checker.

There is a lack of clinical evidence to support these findings on the DDI-checker.

### Methadone

**Figure 4.**
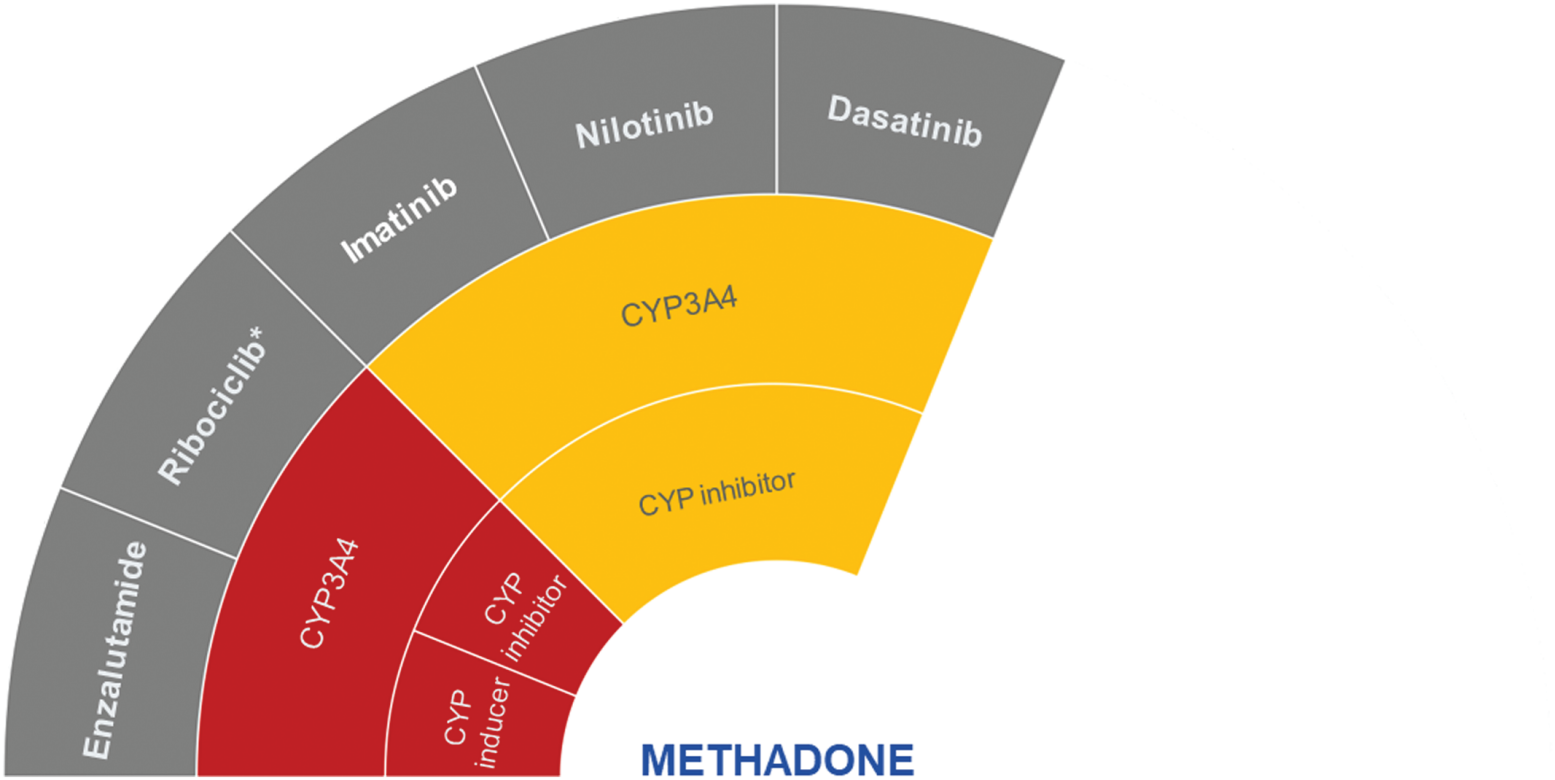
Proposed DDIs between methadone (center) and selected anticancer drugs (gray) categorized as “should not be coadministered” (red) or ‘potentially clinically significant’ (amber) according to the DDI-checker. The inner rings indicate relevant enzymes and transporters involved and whether anticancer drugs act as inhibitors or inducers. Abbreviation: CYP, cytochrome *P450*. *Coadministration of ribociclib with methadone is contraindicated due to QT prolongation.

Methadone is mainly metabolized through demethylation by CYP2B6 and also via CYP3A4.^43,44^ According to the DDI-checker, enzalutamide, ribociclib, imatinib, dasatinib, and nilotinib potentially have clinically significant interactions with methadone (Figure 4).^21^

Enzalutamide should not be coadministered with methadone. Its strong CYP3A4 induction may significantly reduce methadone concentrations and thereby decrease analgesia.^21^ As a dose-dependent inhibitor of CYP3A4, ribociclib may increase methadone concentrations and is also a QT prolonging agent (linked to the risk of potentially fatal torsades de pointes and arrhythmias^21,45^) which contraindicates coadministration at higher doses.^21^

Methadone concentrations may also increase when coadministered with dasatinib, nilotinib, and imatinib, which are all weak/moderate CYP3A4 inhibitors.^21^ Methadone-related toxicity may cause sedation, respiratory depression, and QT prolongation associated with the risk of potentially fatal torsades de pointes and arrhythmias.^46-49^ Therefore, close monitoring including electrocardiogram (ECG) assessment is recommended,^21^ with dose adjustment where necessary.

There is a lack of clinical studies to support these DDI-checker findings. However, there is a case report of potential DDIs between methadone and gefitinib (Table 1).^34^ A patient with stage IV lung carcinoma on long-term methadone treatment died of respiratory failure less than 2 months after taking gefitinib.^34^

### Morphine

Morphine can be used as such or formed from a prodrug like codeine via O-demethylation catalyzed by CYP2D6.^15^ It is primarily metabolized via glucuronidation by UGT2B7 to the inactive morphine-3-glucuronide and, secondarily, to the active morphine-6-glucuronide, which has superior pharmacological activity and longer half-life compared with morphine.^15,50^ Despite lacking an analgesic effect, morphine-3-glucuronide may display neuroexcitatory effects that may cause allodynia, myoclonus, and seizures in humans.^51^

No clinically significant DDIs between morphine and selected anticancer drugs were identified, neither with the DDI-checker nor in the literature search.^21^

### Oxycodone

Oxycodone is mainly metabolized to the inactive metabolite noroxycodone via CYP3A4 and to a lower degree to the active metabolite oxymorphone via CYP2D6.^15^ Oxymorphone undergoes further metabolism to the inactive metabolite noroxymorphone by UGT2B7 and CYP enzymes.^15^

**Figure 5.**
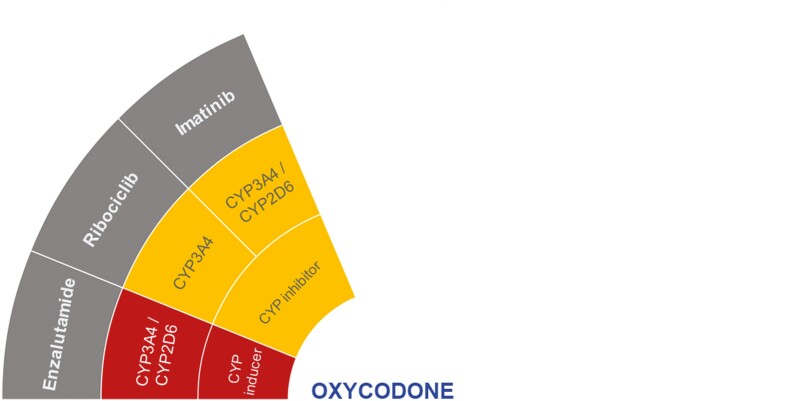
Proposed DDIs between oxycodone (center) and selected anticancer drugs (gray) categorized as “should not be coadministered” (red) or ‘potentially clinically significant’ (amber) according to the DDI-checker. The inner rings indicate relevant enzymes and transporters involved and whether anticancer drugs act as inhibitors or inducers. CYP, cytochrome P450.

The DDI-checker has indicated potential clinically significant interactions of oxycodone with enzalutamide, imatinib, and ribociclib (Figure 5).^21^

Coadministration with enzalutamide should be avoided due to its strong and moderate CYP3A4 and CYP2D6 induction, which could significantly decrease exposure to oxycodone.^21^ In 2 case studies of patients with CRPC, enzalutamide eliminated oxycodone’s analgesic effect, which was resolved by opioid rotation with morphine (Table 1).^33^ Furthermore, in 2 retrospective reviews from 2015 using Lexicomp and Micromedex databases, the first listed enzalutamide and oxycodone coadministration on the category of potential DDI of clinical significance, while this was considered a moderate risk of potential DDI in the second database (Table 1).^36^ In another retrospective review, Lexicomp described the oxycodone and abiraterone interaction as the most flagged clinically significant potential DDI, and in alignment, Micromedex categorized the DDI as a major risk.^37^ In a small prospective study (*N* = 26), patients with prostate cancer received oxycodone with (arm 1) or without enzalutamide (arm 2) (Table 1). Patients in arm 1 had a significant reduction of plasma oxycodone and oxymorphone, compared to those in arm 2.^35^

The DDI-checker identified potential DDIs via ribociclib and imatinib as CYP3A4 inhibitors and CYP2D6 enzymes that could cause oxycodone- and oxymorphone-related toxicity (Figure 5).^21^ However, this could not be verified from literature.

### Tramadol

Tramadol is metabolized by CYP2D6 to the more potent analgesic metabolite O-desmethyltramadol, with subsequent *N*-demethylation to *N*-desmethyltramadol.^15,51,52^

**Figure 6.**
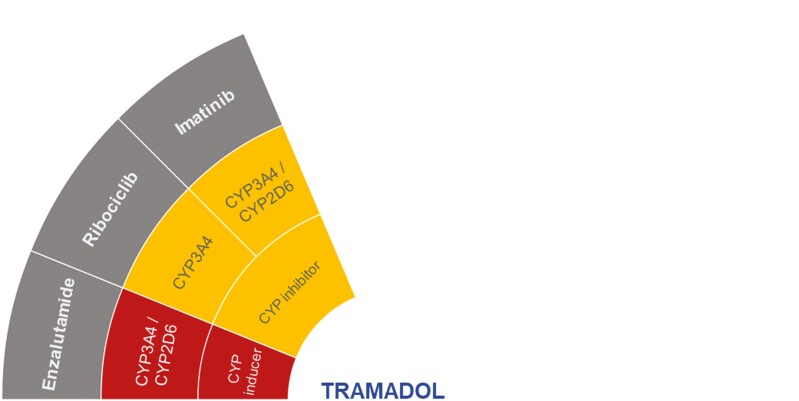
Proposed DDIs between tramadol (center) and selected anticancer drugs (gray) categorized as “should not be coadministered” (red) or “potentially clinically significant” (amber) according to the DDI-checker. The inner rings indicate relevant enzymes and transporters involved and whether anticancer drugs act as inhibitors or inducers. Abbreviation: CYP, cytochrome *P450*.

Further *N*-desmethyltramadol metabolism by CYP2B6 and CYP3A4 forms 3 metabolites that are eventually inactivated by glucuronidation.^52^ This complex metabolism of tramadol and its metabolites creates potential for DDIs with drugs that are CYP inducers or inhibitors, eg, enzalutamide, imatinib, and ribociclib (Figure 6).^15,21,52^

According to the DDI-checker, the CYP3A4/CYP2D6 inducer, enzalutamide, may significantly decrease tramadol concentrations, while weak inhibition of CYP2B6 may increase concentrations of tramadol. Despite this contradiction, efficacy and toxicity must be closely monitored.^21^ In a retrospective DDI assessment of enzalutamide, abiraterone, and apalutamide using the Liverpool and Uptodate (Lexicomp) databases, it was found that although enzalutamide had the highest number of potential DDIs (55.6%) that may alter the treatment’s efficacy and/or safety, the study warned against coadministration of tramadol with abiraterone or apalutamide,^38^ rather than enzalutamide (Table 1). Caution for enzalutamide DDIs with opioids was not provided.^38^

Tramadol concentrations may increase through imatinib and ribociclib as CYP3A4 inhibitors, as well as imatinib’s weak inhibition of CYP2D6. Hence, coadministration of imatinib/ribociclib with tramadol requires close monitoring for tramadol-related toxicity according to the DDI-checker.^21^ Despite the lack of clinical evidence, imatinib seems to be the only selected anticancer drug which aligns with observations from the DDI-checker and review articles,^52,53^ with one of the review articles describing the potential tramadol-related toxicity to possibly include serotonin syndrome or seizure.^52^

## Discussion

This narrative review used a DDI-checker tool to assess potential DDIs resulting from the opioids used for treatment of moderate-to-severe CRP with the concomitant use of common anticancer agents. The findings were compared to observations from a literature search conducted in Embase and PubMed to identify clinical evidence for these potential DDIs, which were limited and mainly comprised of case studies and retrospective reviews.

The majority of identified potential opioid DDIs on the DDI-checker were related to their CYP metabolism, mainly including the inhibition or induction of CYP3A4,^21^ which is consistent with findings from a previous review.^18^ Opioids metabolized by CYP3A4 have a high risk for DDIs as it accounts for the metabolism of ~50% of all available drugs.^15,51^

As a strong inducer of CYP3A4, enzalutamide predominated potential opioid DDIs on the DDI-checker, with a precaution that it should not be coadministered with opioids that are mainly metabolized by CYP3A4, which forms the majority of opioids discussed in this review.^21^ CYP3A4 induction by enzalutamide showed contrasting effects on opioids, with potential toxicity of buprenorphine and hydrocodone, while a potential decrease of analgesia was highlighted with fentanyl, methadone, oxycodone, and tramadol, according to the DDI-checker. Despite the lack of supporting clinical evidence for most of these opioids, small comparative/prospective studies and case studies have reported that the concomitant use of enzalutamide with CYP3A4-metabolized opioids should be avoided.^32,33,36,54^ In some of these studies, opioid rotation to a non-CYP3A4-metabolized opioid, such as morphine, was recommended.^32,33,35^

As morphine is not extensively metabolized by CYP enzymes, DDIs associated with it are considered rare.^30^ It is the only opioid to show no risk of potential clinically significant DDIs on the DDI-checker and in clinical studies.^21^ In addition to morphine, non-CYP3A4-metabolized opioids such as hydromorphone and oxymorphone may be preferred options in drug interactions involving investigational novel drugs in early phase clinical trials, which are not captured on DDI-checker tools. Routes of administration and formulations of opioids that avoid first-pass extraction could also represent an alternative strategy to mitigate the risk of clinically relevant DDIs.^55^

In contrast to morphine, methadone is extensively metabolized by CYP enzymes,^56^ and was identified on the DDI-checker as being most prone to clinically significant DDIs with selected anticancer drugs compared to other opioids.^21^ This is despite the lack of clinical evidence to support this, with only one case study describing a patient that had received methadone for over 30 years, suddenly developed tachypnea and then died from respiratory failure <2 months after coadministration with gefitinib.^34^ Gefitinib is associated with interstitial pneumonia,^57^ and respiratory depression is the most serious adverse event of treatment with methadone.^34^ Thus, the case study cautioned coadministration of methadone with gefinitib and advised HCPs to pay attention to clinical respiratory symptoms.^34^ Methadone is also a substrate of P-gp during first-pass metabolism, where P-gp inhibitors may increase its transport across the intestinal wall or blood-brain barrier.^52,58^ Therefore, coadministration with P-gp inhibitors like nilotinib must be avoided according to a review article,^52^ which however lacks clinical evidence.

Although none of the potential clinically significant opioid DDIs were associated with P-gp on the DDI-checker,^21^ these were mentioned in a review article in association with opioids such as fentanyl, hydrocodone, morphine, and methadone, when coadministered with P-gp inhibitors like nilotinib,^52^ despite lacking clinical evidence. There are more examples of potential DDIs found in the literature that were considered clinically insignificant on the DDI-checker, eg, oxycodone DDIs with tamoxifen,^52^ nilotinib and dasatinib,^52,53^ as well as tramadol DDIs with dasatinib, nilotinib, and gefitinib.^52,53^

Similarly, there are potential DDIs that were undetected on the DDI-checker but were mentioned in the SmPCs of CYP3A4 inhibitors such as nilotinib, imatinib, and ribociclib, which cautioned against coadministration with drugs with a narrow therapeutic window such as fentanyl.^29,59,60^

Notably, no clinically significant DDIs between opioids and selected biologics were proposed by the DDI-checker,^21^ and none were found in the literature search.

The current study documents tool-informed assessments of opioid DDIs and their clinical implications in patients with cancer. This is important as the DDI-checker website is set to go offline in September 2024 due to a lack of sustainable long-term funding, according to the University of Liverpool.^21^ Relevant institutions may need to consider prioritizing the maintenance of such checker tools, as their existence and accessibility may help to minimize potential health risks associated with unfavorable DDIs.

### Expert opinion

Internet checker tools and databases can help HCPs identify clinically relevant potential DDIs with opioids in patients with cancer. Research has demonstrated that, without software aids, the HCP’s ability to identify well documented and even clinically significant DDIs is limited.^61,62^ Unfortunately, existing systems are far from fail-safe as evidence indicates that HCPs may still miss clinically important DDIs, particularly in patients with polypharmacy.^63^ The majority of checker tools analyze DDIs in a 1:1 drug pairwise manner.^64,65^ For patients with polypharmacy, pairwise analysis often generates multiple incoherent results, leaving HCPs uninformed about the most probable and serious consequences of the interactions and unsure of the precise interventions needed to appropriately mitigate these interactions.^65^

Proposed DDIs from these platforms do not necessarily reflect the clinical relevance of interactions as they are mostly based on PK and PD studies. This may affect the choice of molecule, dose, and duration of treatment. Direct source of information is often not referenced on checker tools; thus, they are hard to verify. The general lack of clinical data to verify DDI-checker findings is largely attributed to ethical limitations that may challenge randomized controlled trials or other well-designed clinical studies based on opioid DDIs.^18,66^ Furthermore, checker tools and databases may lack ranking in reliability, which pose a challenge when the severity of a potential DDI varies between these platforms. Lastly, not every drug is available on the platforms (eg, platinum agents are absent on the DDI-checker).^21^

In light of these limitations, HCPs must take necessary precautions to prioritize safe and effective treatment of the patient when utilizing the aid of these platforms. This entails HCPs verifying their reliability through research of reported validation in peer reviewed journals, confirming if principles of evidence-based medicine are applied and if endorsement by professional organizations and national medicine agencies was provided. HCPs are also advised to select the worst classification of a potential DDI when the severity varies between different platforms.

Utilizing a standardized data exchange model is of the utmost importance. When there are no accredited standards, a multifaceted technological overhaul to address the identified content and system-related shortcomings is required. Ideally, checker tools must be readily accessible, user friendly, on-demand/timely, interactive with medical and drug information, up-to-date, and secure (by adhering to privacy compliance), and should also be integrated into hospital workflows and programs.

Software applications may be used to develop a checker tool that can be integrated in electronic prescription service (EPS) and electronic health record (EHR) programs, that can display pop-up warnings in case of potential DDIs. Development of uniform EPS and EHR programs for patients across different health systems and pharmacies could improve detection of potential DDIs. An ideal drug interaction alert software would analyze simultaneous multidrug interactions and comprehensively describe the mechanisms and severity underlying these interactions. This would enable HCPs to easily and efficiently assess interaction risks using risk prediction models,^67,68^ in order to optimize pain management and patients’ overall healthcare.^69^

While checker tools may provide guidance in the decision-making process, this should not replace the need for clinical assessment by experts when optimizing the use of opioids. The clinical relevance of a potential DDI may require a multidisciplinary team to ensure accurate interpretation of DDI information gathered from checker tools. However, such collaboration between clinical pharmacologists and treating physicians is available in large hospitals and academic centers, but not very common in the developing countries.

A holistic approach to the management of CRP that does not solely rely on drugs should always be considered when navigating DDIs. The intersection of the biological, psychological, and social aspects of a patient’s life is where their overall wellbeing lies, thus warranting a patient-centered approach to all DDI concerns. Some DDIs might be more significant based on the status of certain organ functions (eg, renal clearance, hepatic function, or short bowel), patient’s genetic composition (eg, genetically determined CYP2D6 activity, with patients exhibiting different metabolizer phenotypes),^70^ patient’s sex (eg, lipophilic drugs exhibit prolonged action in fatty tissue, which is relevant for most females who usually have a higher percentage of body fat) and specific populations (eg, QT prolongers, older patients or patients with late-stage cancer). For example, in end-of-life palliative care, comfort of patients is prioritized and thresholds to consider DDIs are lower. Therefore, HCPs must always deploy clinical judgement, monitoring, and of course documentation of every clinical scenario involving DDIs.

It is crucial to consider the multifaceted nature of DDIs and the uniqueness of each patient when HCPs make treatment decisions informed with the aid of checker tools and/or databases. Although these are very valuable tools, their results need to be validated and they will need further improvement until their recommendations are integrated in national and international clinical practice guidelines.

## Conclusion

A DDI-checker tool was used to put the outcome of tool-informed assessments of DDIs into context with clinical implications and practice. The findings were compared to observations from literature search results, which were limited and mainly comprised of case studies and retrospective reviews. Although some potential DDIs observed on the DDI-checker were aligned with literature findings (eg, concomitant use of enzalutamide with CYP3A4-metabolized opioids must be avoided), there were also potential DDIs found in literature that were considered clinically insignificant (eg, tamoxifen- and oxycodone-related toxicity) or absent (eg, P-gp inhibitors like nilotinib interacting with opioids) on the DDI-checker. Therefore, irrespective of whether there are potential DDIs identified on a DDI checker, patients should be continuously monitored, and benefits and risks weighed. HCPs must consider these outcomes with caution and use a holistic approach of thorough clinical assessment of the patient, literature verification, and multidisciplinary team collaboration before implementing tool-informed decisions in clinical practice. To ensure their continuous value, DDI checker tools require ongoing maintenance with up-to-date clinical evidence and experience.

## Supplementary material

Supplementary material is available at *The Oncologist* online.

oyae094_suppl_Supplementary_Materials

## Data Availability

The data underlying this article are available in an open access interaction checker website (Cancer—Drug interactions checker) at https://cancer-druginteractions.org/checker until September 2024.
