# Supplementary material for: Opioid metabolism and drug-drug interaction in cancer
Source: Oncologist. 2024 May 23;29(11):931–42. doi: 10.1093/oncolo/oyae094 (PMC11546622; doi:10.1093/oncolo/oyae094)
Supplement: oyae094_suppl_Supplementary_Materials [file oyae094_suppl_supplementary_materials.docx]

# Supplementary data 1: Drug-drug interaction (DDI) checker

## Quality assurance of DDI checker

Treatment recommendations are made based on available evidence and the quality of this evidence must be assessed. For the current study, the strength of recommendation of an opioid DDI with specific cancer treatment was indicated by a drug interaction chart (i.e., red, amber, yellow, green). The quality of evidence behind that recommendation was graded from high to very low.

## Evaluation of the quality of evidence

The Grading of Recommendations, Assessment, Development, and Evaluation (GRADE) was used for rating evidence and recommendations. The working group for the GRADE system is represented in many organizations including the Agency for Healthcare Research and Quality in the US, the National institute for Clinical Excellence for England and Wales, and the World Health Organization (WHO). The background and workings of the GRADE system is described in the following articles:

- What is “quality of evidence” and why is it important to clinicians?
  Guyatt et al. *BMJ* 2008; 336 doi: <https://doi.org/10.1136/bmj.39490.551019.BE>
- GRADE: an emerging consensus on rating quality of evidence and strength of recommendations.
  Guyatt et al. *BMJ* 2008; 336 doi: <https://doi.org/10.1136/bmj.39489.470347.AD>
- Grading quality of evidence and strength of recommendations.

Atkins et al. *BMJ* 2004; 328 doi: <https://doi.org/10.1136/bmj.328.7454.1490>

Table S1: Criteria used to determine the quality of evidence when assessing interaction data on the DDI checker, adapted from the DDI checker website (<https://cancer-druginteractions.org/checker>).


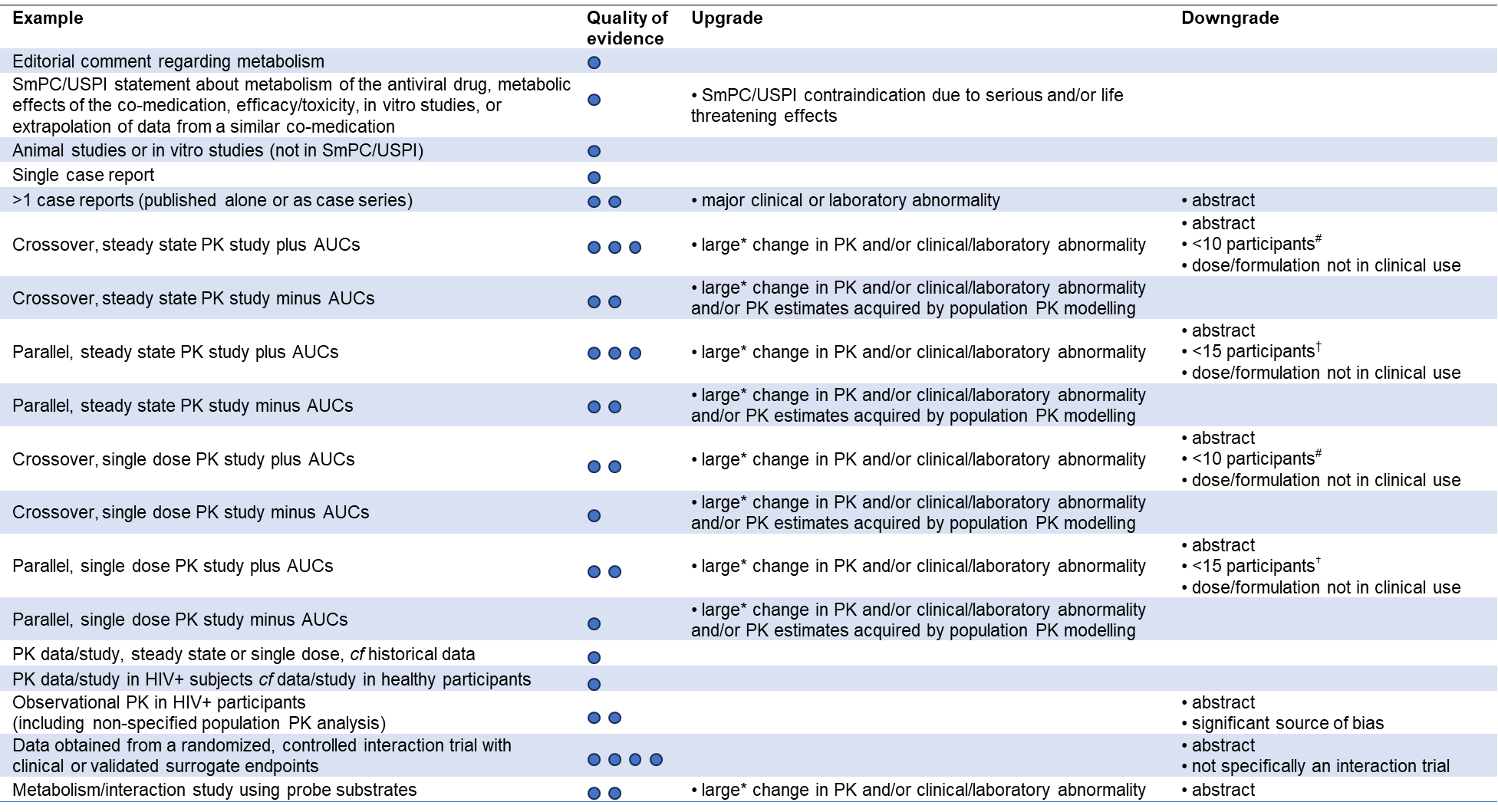


#
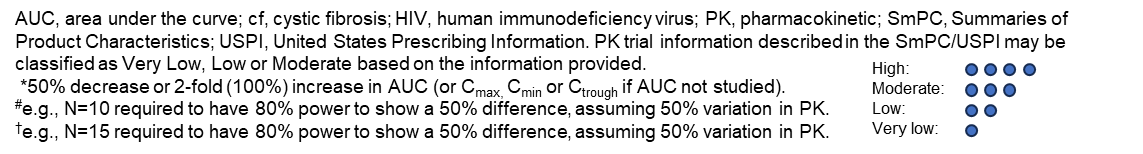


# Supplementary data 2: Search strategy and data extraction

## Search strategy

A literature search was conducted in Embase and PubMed from January 1990 until March 2023 to identify clinical evidence for Drug-drug interactions (DDIs) expected for each pair of opioid and anticancer drugs. Randomized controlled trials (RCTs), observational studies, retrospective studies, case reports, abstracts, and congress data were included. Search terms included frequently used opioids for moderate-to-severe cancer-related pain (CRP) (1), and selected anticancer drugs for treatment of common types of cancer such as female breast, lung, and prostate cancers.(2, 3) The search strategy involved developing strings of terms and synonyms to capture three core factors in the review:

1. **Opioids**: buprenorphine, fentanyl, hydrocodone, methadone, morphine, oxycodone, and tramadol.
2. **Anticancer drugs**: chemotherapeutics (taxanes, specifically paclitaxel and docetaxel; platinum agents, specifically oxaliplatin, carboplatin, and cisplatin; oxazophosphorines, specifically cyclophosphamide; and vinca alkaloids, specifically vincristine), tyrosine kinase inhibitors (dasatinib, gefitinib, imatinib, nilotinib), biologics (atezolizumab, nivolumab, trastuzumab), cyclin-dependent kinase 4/6 inhibitors (palbociclib, ribociclib), estrogen receptor modulators (tamoxifen), steroid hormones (abiraterone), and nonsteroidal antiandrogens (enzalutamide).
3. **DDI terms**: drug interaction(s), drug-drug interaction(s), drug antagonism, polypharmacy, drug synergism(s), drug potentiation(s), drug augmentation(s), drug toxicity, drug competition, drug inhibition, drug intoxication.

Table S2: A search string from Proquest, searched on the Embase platform.

| **Set#** | **Searched for** | **Results** |
| --- | --- | --- |
| S1 | EMB.EXACT.EXPLODE("buprenorphine") OR Buprenorphin OR Buprenorphine OR 6029-M OR 6029M OR "6029 M" OR Buprenex OR Buprex OR Prefin OR Sebutex OR Temgesic | 26697* |
| S2 | EMB.EXACT.EXPLODE("fentanyl") OR Fentanyl OR Duragesic OR Durogesic OR Fentanest OR Fentora OR Phentanyl OR R-4263 OR R4263 OR "R 4263" OR Sublimaze OR Lazanda OR Pecfent | 82265* |
| S3 | EMB.EXACT.EXPLODE("hydromorphone") OR Hydromorphone OR Dihydromorphinone OR Dilaudid OR Hydromorphon OR Laudacon OR Palladone OR Himop OR Sophidone | 12993* |
| S4 | EMB.EXACT.EXPLODE("hydrocodone") OR EMB.EXACT.EXPLODE("hydrocodone bitartrate") OR hydrocodone OR Codinovo OR Dicodid OR Dihydrocodeinone OR Hycodan OR Hycon OR Hydrocodon OR Robidone OR Cardiazol OR Dalmacol | 9598* |
| S5 | EMB.EXACT.EXPLODE("methadone") OR methadon OR methadone OR Amidone OR Dolophine OR Metadol OR Methaddict OR Methadose OR Methex OR Phenadone OR Phymet OR Physeptone OR Symoron OR Rubidexol | 41657* |
| S6 | EMB.EXACT.EXPLODE("morphine") OR morphine OR morphin OR Duramorph OR Morphia OR Oramorph | 142594* |
| S7 | EMB.EXACT.EXPLODE("tramadol") OR tramadol OR tramadole OR Adolonta OR Amadol OR Biodalgic OR Biokanol OR Contramal OR Jutadol OR K-315 OR K315 OR "K 315" OR Nobligan OR Prontofort OR Takadol OR Theradol OR Tiral OR Topalgic OR Tradol OR Tradonal OR Tralgiol OR Tramadin OR Tramadoc OR Tramadolor OR Tramadura OR Tramagetic OR Tramagit OR Tramake OR Tramal OR Tramex OR Tramundin OR Trasedal OR Ultram OR Zamudol OR Zumalgic OR Zydol OR Zytram OR Tramal | 29004* |
| S8 | EMB.EXACT.EXPLODE("oxycodone") OR oxycodone OR Dihydrohydroxycodeinone OR Dihydrone OR Dinarkon OR Eucodal OR Oxiconum OR Oxycodeinon OR Oxycone OR Oxycontin OR Pancodine OR Theocodin OR Xtampza | 24170* |
| S9 | S8 OR S7 OR S6 OR S5 OR S4 OR S3 OR S2 OR S1 | 272221* |
| S10 | EMB.EXACT.EXPLODE("Abiraterone") OR EMB.EXACT.EXPLODE("Abiraterone Acetate") OR Abiraterone OR CB7598 OR CB 7598 OR CB-7598 OR JNJ 212082 OR JNJ-212082 OR JNJ212082 OR Zytiga | 9959* |
| S11 | EMB.EXACT.EXPLODE("atezolizumab") OR atezolizumab OR L01XC32 OR MDPL 3280A OR MDPL-3280A OR MDPL3280A OR MPDL 3280A OR MPDL 328OA OR MPDL-3280A OR MPDL-328OA OR MPDL3280A OR MPDL328OA OR RG 7446 OR RG-7446 OR RG7446-42 OR RG7446 OR RO 5541267 OR RO-5541267 OR RO5541267 OR Tecentriq | 14228* |
| S12 | EMB.EXACT.EXPLODE("cyclophosphamide") OR EMB.EXACT.EXPLODE("cyclophosphamide derivative") OR cyclophosphamide OR cyclophosphamid OR Cyclophosphane OR Cytophosphan OR Cytophosphane OR Cytoxan OR Endoxan OR NSC-26271 OR NSC26271 OR "NSC 26271" OR Neosar OR Procytox OR Sendoxan | 264932* |
| S13 | EMB.EXACT.EXPLODE("dasatinib") OR dasatinib OR "BMS 354825" OR BMS-354825 OR BMS354825 OR Sfriisl OR Spricel OR Sprycel | 17714* |
| S14 | EMB.EXACT.EXPLODE("enzalutamide") OR enzalutamide OR "ASP 9785" OR ASP-9785 OR ASP9785 OR "MDV 3100" OR MDV-3100 OR MDV3100 OR Xtandi OR "HC 119" OR HC119 OR HC-119 | 9879* |
| S15 | EMB.EXACT.EXPLODE("gefitinib") OR gefitinib OR Iressa OR "ZD 1839" OR ZD-1839 OR ZD1839 | 29573* |
| S16 | "Imatinib mesylate" OR EMB.EXACT.EXPLODE("imatinib") OR Ciplevac OR Imatib OR Imavec OR CGP57148 OR "CGP 57148" OR CGP-57148 OR "ST 1571" OR ST-1571 OR ST1571 | 48804* |
| S17 | EMB.EXACT.EXPLODE("nilotinib") OR nilotinib OR "AMN 107" OR AMN-107 OR AMN107 OR Tasigna | 11468* |
| S18 | EMB.EXACT.EXPLODE("nivolumab") OR nivolumab OR "BMS 936558" OR "BMS 986298" OR BMS-936558 OR BMS-986298 OR BMS936558 OR BMS986298 OR "MDX 1106" OR MDX-1106 OR MDX1106 OR "ONO 4538" OR ONO-4538 OR ONO4538 OR Opdivo OR Opdyta | 35995* |
| S19 | EMB.EXACT.EXPLODE("palbociclib") OR palbociclib OR Ibrance OR Itulsi OR "PD 0332991" OR "PD 991" OR PD-0332991 OR PD-991 OR PD0332991 OR PD991 | 6455* |
| S20 | EMB.EXACT.EXPLODE("ribociclib") OR ribociclib OR Kisqali OR Kryxana OR "LEE 011 A" OR "LEE 011" OR "LEE 011A" OR LEE-011-A OR LEE-011 OR LEE-011A OR LEE011 A OR LEE011-A OR LEE011-BBA OR LEE011 OR LEE011A | 2548° |
| S21 | EMB.EXACT.EXPLODE("trastuzumab") OR trastuzumab OR Herceptin OR Herclon OR "RG 597" OR RG-597 OR RG597 OR Trazimera | 50317* |
| S22 | EMB.EXACT.EXPLODE("vincristine") OR EMB.EXACT.EXPLODE("vincristine sulfate") OR vincristine OR vincristin OR Citomid OR Farmistin OR Leurocristine OR Oncovin OR Oncovine OR Onkocristin OR Vincasar OR Vincrisul OR Vintec OR cellcristin OR Fauldvincri | 124391* |
| S23 | EMB.EXACT.EXPLODE("paclitaxel") OR paclitaxel OR Anzatax OR NSC-125973 OR Onxol OR Paxene OR Praxel OR Taxol OR NSC125973 OR "NSC 125973" OR Apealea OR Paclical | 137085* |
| S24 | EMB.EXACT.EXPLODE("docetaxel") OR docetaxel OR Docetaxol OR "NSC 628503" OR "RP 56976" OR RP-56976 OR Taxoltere OR Taxotere OR NSC-628503 OR NSC628503 OR RP56976 | 73467* |
| S25 | EMB.EXACT.EXPLODE("cisplatin") OR EMB.EXACT.EXPLODE("cisplatin derivative") OR cisplatin OR cisplatine OR Biocisplatinum OR Dichlorodiammineplatinum OR NSC-119875 OR "NSC 119875" OR NSC119875 OR Platidiam OR Platino OR Platinol OR "Platinum Diamminodichloride" OR cis-Diamminedichloroplatinum OR cis-Platinum OR "cis Diamminedichloroplatinum" OR "cis Platinum" | 224459* |
| S26 | EMB.EXACT.EXPLODE("carboplatin") OR carboplatin OR carboplatine OR Blastocarb OR CBDCA OR Carboplat OR Carbosin OR Carbotec OR Ercar OR JM-8 OR JM8 OR "JM 8" OR NSC-241240 OR NSC241240 OR "NSC 241240" OR Nealorin OR Neocarbo OR Paraplatin OR Paraplatine OR Platinwas OR Ribocarbo | 87152* |
| S27 | EMB.EXACT.EXPLODE("oxaliplatin") OR oxaliplatin OR oxaliplatine OR "ACT 078" OR ACT-078 OR ACT078 OR Eloxatin OR Eloxatine OR Dacotin OR Elplat | 53704* |
| S28 | (EMB.EXACT.EXPLODE("tamoxifen") OR tamoxifen OR tamoxifene OR ICI-46,474 OR ICI-46474 OR ICI-47699 OR "ICI 47699" OR ICI47699 OR Nolvadex OR Novaldex OR Soltamox OR Tomaxithen OR Zitazonium) | 76107* |
| S29 | S28 OR S27 OR S26 OR S25 OR S24 OR S23 OR S22 OR S21 OR S20 OR S19 OR S18 OR S17 OR S16 OR S15 OR S14 OR S13 OR S12 OR S11 OR S10 | 839257* |
| S30 | S29 AND S9 | 5108* |
| S31 | (S29 AND S9) and (pd(19900101-20231231)) | 4775° |
| S32 | EMB.EXACT.EXPLODE("drug interaction") OR “Drug-drug Interactions” OR “Drug Interactions” OR “Drug Interaction” OR “Drug Antagonism” OR “Polypharmacy” OR “Drug Synergism” OR “Drug Synergisms” OR “Drug Potentiation” OR “Drug Potentiations” OR “Drug Augmentation” OR “Drug Augmentations” OR “Drug interaction” OR “Drug interactions” OR “Drug toxicity” OR “Drug toxicities” OR “Drug competition” OR “Drug inhibition” OR “Drug intoxication” | 1201624* |
| S33 | S32 AND S31 | 914° |
| S34 | (Ti,ab((Buprenorphine OR Fentanyl OR Hydromorphone OR Hydrocodone OR Methadone OR Morphine OR Oxycodone OR Tramadol) AND (Abiraterone OR Atezolizumab OR Cyclophosphamide OR Dasatinib OR Enzalutamide OR Gefitinib OR Imatinib OR nilotinib OR nivolumab OR palbociclib OR ribociclib OR tamoxifen OR trastuzumab OR vincristine OR paclitaxel OR docetaxel OR cisplatin OR carboplatin OR oxaliplatin) AND (“Drug-drug Interactions” OR “Drug Interactions” OR “Drug Antagonism” OR “Drug Synergism” OR Polypharmacy OR “Drug Synergism” OR “Drug Synergisms” OR “Drug Potentiation” OR “Drug Potentiations” OR “Drug Augmentation” OR “Drug Augmentations” OR “Drug interaction” OR “Drug toxicity” OR “Drug competition” OR “Drug inhibition” OR “Drug intoxication”))) and (pd(>19900101)) | 21° |
| S35 | Ti,ab(S9) | 145543* |
| S36 | Ti,ab(S29) | 432874* |
| S37 | Ti,ab(S32) | 77442* |
| S38 | S37 AND S36 AND S35 | 24° |
| S39 | (S37 AND S36 AND S35) and (pd(19900101-20231231)) | 23° |
| S40 | (S34 NOT S39) | 0° |
| S41 | (S37 AND S36 AND S35) and (rtype.exact("Conference Abstract" OR "Conference Review") AND pd(19900101-20231231)) | 8° |
| S42 | (S39 NOT S41) | 15° |
| S43 | (S1 AND S39) | 2° |
| S44 | (S2 AND S39) | 3° |
| S45 | (S3 AND S39) | 1° |
| S46 | (S4 AND S39) | 1° |
| S47 | (Ti,ab(S1) AND S39) | 2° |
| S48 | (Ti,ab(S2) AND S39) | 3° |
| S49 | (Ti,ab(S1)) AND S39 | 2° |
| S50 | (Ti,ab(S2)) AND S39 | 3° |
| S51 | (Ti,ab(S3)) AND S39 | 0° |
| S52 | ((Ti,ab(S4)) AND S39) | 0° |
| S53 | ((Ti,ab(S5)) AND S39) | 9° |
| S54 | ((Ti,ab(S6)) AND S39) | 5° |
| S55 | ((Ti,ab(S7)) AND S39) | 4° |
| S56 | ((Ti,ab(S8)) AND S39) | 8° |
| S57 | ((Ti,ab(S11)) AND S39) | 0° |
| S58 | (((Ti,ab(S12)) AND S39)) | 3° |
| S59 | (((Ti,ab(S13)) AND S39)) | 0° |
| S60 | ((((Ti,ab(S14)) AND S39))) | 4° |
| S61 | ((((Ti,ab(S15)) AND S39))) | 1° |
| S62 | ((((Ti,ab(S16)) AND S39))) | 4° |
| S63 | ((((Ti,ab(S17)) AND S39))) | 0° |
| S64 | (((((Ti,ab(S18)) AND S39)))) | 0° |
| S65 | (((((Ti,ab(S19)) AND S39)))) | 1° |
| S66 | (((((Ti,ab(S20)) AND S39)))) | 0° |
| S67 | ((((((Ti,ab(S21)) AND S39))))) | 0° |
| S68 | ((((((Ti,ab(S22)) AND S39))))) | 3° |
| S69 | (Ti,ab(S23)) AND S39 | 1° |
| S70 | (Ti,ab(S24)) AND S39 | 1° |
| S71 | (Ti,ab(S25)) AND S39 | 3° |
| S72 | (Ti,ab(S26)) AND S39 | 1° |
| S73 | (Ti,ab(S27)) AND S39 | 0° |
| S74 | ((Ti,ab(S28)) AND S39) | 5° |
| S75 | Ti,ab(S27) | 23238* |
| S76 | Ti,ab(oxaliplatin OR oxaliplatine OR "ACT 078" OR ACT-078 OR ACT078 OR Eloxatin OR Eloxatine OR Dacotin OR Elplat) | 23238* |

Table S3: A search string from Proquest, searched on the PubMed platform.

| **Search** | **Query** | **Results** |
| --- | --- | --- |
| #1 | Search: "Buprenorphine"[MeSH] OR "Buprenorphin"[All] OR "Buprenorphine"[All] OR "6029-M"[All] OR "6029M"[All] OR "6029 M"[All] OR "Buprenex"[All] OR "Buprex"[All] OR "Prefin"[All] OR "Sebutex"[All] OR "Temgesic"[All] Sort by: Publication Date | 10,095 |
| #2 | Search: "Fentanyl"[MeSH] OR "Fentanyl"[All] OR "Duragesic"[All] OR "Durogesic"[All] OR "Fentanest"[All] OR "Fentora"[All] OR "Phentanyl"[All] OR "R-4263"[All] OR "R4263"[All] OR "R 4263"[All] OR "Sublimaze"[All] OR "Lazanda"[All] OR "Pecfent"[All] Sort by: Publication Date | 27,853 |
| #3 | Search: "Hydromorphone"[MeSH] OR "Hydromorphone"[All] OR "Dihydromorphinone"[All] OR "Dilaudid"[All] OR "Hydromorphon"[All] OR "Laudacon"[All] OR "Palladone"[All] OR "Himop"[All] OR "Sophidone"[All] Sort by: Publication Date | 2,523 |
| #4 | Search: "Hydrocodone"[MeSH] OR "hydrocodone"[All] OR "Codinovo"[All] OR "Dicodid"[All] OR "Dihydrocodeinone"[All] OR "Hycodan"[All] OR "Hycon"[All] OR "Hydrocodon"[All] OR "Robidone"[All] OR "Cardiazol"[All] OR "Dalmacol"[All] Sort by: Publication Date | 1,748 |
| #5 | Search: "Methadone"[MeSH] OR "methadon"[All] OR "methadone"[All] OR "Amidone"[All] OR "Dolophine"[All] OR "Metadol"[All] OR "Methaddict"[All] OR "Methadose"[All] OR "Methex"[All] OR "Phenadone"[All] OR "Phymet"[All] OR "Physeptone"[All] OR "Symoron"[All] OR "Rubidexol"[All] Sort by: Publication Date | 18,742 |
| #6 | Search: "Morphine"[MeSH] OR "morphine"[All] OR "morphin"[All] OR "Duramorph"[All] OR "Morphia"[All] OR "Oramorph"[All] Sort by: Publication Date | 64,125 |
| #7 | Search: "Oxycodone"[MeSH] OR "oxycodone"[All] OR "Dihydrohydroxycodeinone"[All] OR "Dihydrone"[All] OR "Dinarkon"[All] OR "Eucodal"[All] OR "Oxiconum"[All] OR "Oxycodeinon"[All] OR "Oxycone"[All] OR "Oxycontin"[All] OR "Pancodine"[All] OR "Theocodin"[All] OR "Xtampza"[All] Sort by: Publication Date | 5,183 |
| #8 | Search: "Tramadol"[MeSH] OR "tramadol"[All] OR "tramadole"[All] OR "Adolonta"[All] OR "Amadol"[All] OR "Biodalgic"[All] OR "Biokanol"[All] OR "Contramal"[All] OR "Jutadol"[All] OR "K-315"[All] OR "K315"[All] OR "K 315"[All] OR "Nobligan"[All] OR "Prontofort"[All] OR "Takadol"[All] OR "Theradol"[All] OR "Tiral"[All] OR "Topalgic"[All] OR "Tradol"[All] OR "Tradonal"[All] OR "Tralgiol"[All] OR "Tramadin"[All] OR "Tramadoc"[All] OR "Tramadolor"[All] OR "Tramadura"[All] OR "Tramagetic"[All] OR "Tramagit"[All] OR "Tramake"[All] OR "Tramal"[All] OR "Tramex"[All] OR "Tramundin"[All] OR "Trasedal"[All] OR "Ultram"[All] OR "Zamudol"[All] OR "Zumalgic"[All] OR "Zydol"[All] OR "Zytram"[All] OR "Tramal"[All] Sort by: Publication Date | 6,766 |
| #9 | Search: #1 OR #2 OR #3 OR #4 OR #5 OR #6 OR #7 OR #8 Sort by: Publication Date | 118,093 |
| #10 | Search: "Abiraterone"[Supplementary Concept] OR "Abiraterone"[All] OR "CB7598"[All] OR "CB 7598"[All] OR "CB-7598"[All] OR "JNJ 212082"[All] OR "JNJ-212082"[All] OR "JNJ212082"[All] OR "Zytiga"[All] Sort by: Publication Date | 3,153 |
| #11 | Search: "Atezolizumab"[Supplementary Concept] OR "atezolizumab"[All] OR "L01XC32"[All] OR "MDPL 3280A"[All] OR "MDPL-3280A"[All] OR "MDPL3280A"[All] OR "MPDL 3280A"[All] OR "MPDL 328OA"[All] OR "MPDL-3280A"[All] OR "MPDL-328OA"[All] OR "MPDL3280A"[All] OR "MPDL328OA"[All] OR "RG 7446"[All] OR "RG-7446"[All] OR "RG7446-42"[All] OR "RG7446"[All] OR "RO 5541267"[All] OR "RO-5541267"[All] OR "RO5541267"[All] OR "Tecentriq"[All] Sort by: Publication Date | 2,836 |
| #12 | Search: "Cyclophosphamide"[MeSH] OR "cyclophosphamide"[All] OR "cyclophosphamid"[All] OR "Cyclophosphane"[All] OR "Cytophosphan"[All] OR "Cytophosphane"[All] OR "Cytoxan"[All] OR "Endoxan"[All] OR "NSC-26271"[All] OR "NSC26271"[All] OR "NSC 26271"[All] OR "Neosar"[All] OR "Procytox"[All] OR "Sendoxan"[All] Sort by: Publication Date | 80,301 |
| #13 | Search: "Dasatinib"[MeSH] OR "dasatinib"[All] OR "BMS 354825"[All] OR "BMS-354825"[All] OR "BMS354825"[All] OR "Sfriisl"[All] OR "Spricel"[All] OR "Sprycel"[All] Sort by: Publication Date | 4,481 |
| #14 | Search: "Enzalutamide"[Supplementary Concept] OR "enzalutamide"[All] OR "ASP 9785"[All] OR "ASP-9785"[All] OR "ASP9785"[All] OR "MDV 3100"[All] OR "MDV-3100"[All] OR "MDV3100"[All] OR "Xtandi"[All] OR "HC 119"[All] OR "HC119"[All] OR "HC-119"[All] Sort by: Publication Date | 3,093 |
| #15 | Search: "Gefitinib"[MeSH] OR "gefitinib"[All] OR "Iressa"[All] OR "ZD 1839"[All] OR "ZD-1839"[All] OR "ZD1839"[All] Sort by: Publication Date | 8,527 |
| #16 | Search: "Imatinib mesylate"[MeSH] OR "Imatinib"[All] OR "Ciplevac"[All] OR "Imatib"[All] OR "Imavec"[All] OR "CGP57148"[All] OR "CGP 57148"[All] OR "CGP-57148"[All] OR "ST 1571"[All] OR "ST-1571"[All] OR "ST1571"[All] Sort by: Publication Date | 17,468 |
| #17 | Search: "Nilotinib"[Supplementary Concept] OR "nilotinib"[All] OR "AMN 107"[All] OR "AMN-107"[All] OR "AMN107"[All] OR "Nilotinib"[All] OR "Tasigna"[All] Sort by: Publication Date | 2,740 |
| #18 | Search: "Nivolumab"[MeSH] OR "nivolumab"[All] OR "BMS 936558"[All] OR "BMS 986298"[All] OR "BMS-936558"[All] OR "BMS-986298"[All] OR "BMS936558"[All] OR "BMS986298"[All] OR "MDX 1106"[All] OR "MDX-1106"[All] OR "MDX1106"[All] OR "ONO 4538"[All] OR "ONO-4538"[All] OR "ONO4538"[All] OR "Opdivo"[All] OR "Opdyta"[All] Sort by: Publication Date | 9,235 |
| #19 | Search: "Palbociclib"[Supplementary Concept] OR "palbociclib"[All] OR "Ibrance"[All] OR "Itulsi"[All] OR "PD 0332991"[All] OR "PD 991"[All] OR "PD-0332991"[All] OR "PD-991"[All] OR "PD0332991"[All] OR "PD991"[All] Sort by: Publication Date | 1,765 |
| #20 | Search: "Ribociclib"[Supplementary Concept] OR "ribociclib"[All] OR "Kisqali"[All] OR "Kryxana"[All] OR "LEE 011 A"[All] OR "LEE 011"[All] OR "LEE 011A"[All] OR "LEE-011-A"[All] OR "LEE-011"[All] OR "LEE-011A"[All] OR "LEE011 A"[All] OR "LEE011-A"[All] OR "LEE011-BBA"[All] OR "LEE011"[All] OR "LEE011A"[All] Sort by: Publication Date | 675 |
| #21 | Search: "Tamoxifen"[MeSH] OR "tamoxifen"[All] OR "tamoxifene"[All] OR "ICI-46,474"[All] OR "ICI-46474"[All] OR "ICI-47699"[All] OR "ICI 47699"[All] OR "ICI47699"[All] OR "Nolvadex"[All] OR "Novaldex"[All] OR "Soltamox"[All] OR "Tomaxithen"[All] OR "Zitazonium"[All] Sort by: Publication Date | 32,294 |
| #22 | Search: "Trastuzumab"[MeSH] OR "trastuzumab"[All] OR "Herceptin"[All] OR "Herclon"[All] OR "RG 597"[All] OR "RG-597"[All] OR "RG597"[All] OR "Trazimera"[All] Sort by: Publication Date | 14,639 |
| #23 | Search: "Vincristine"[MeSH] OR "vincristine"[All] OR "vincristin"[All] OR "Citomid"[All] OR "Farmistin"[All] OR "Leurocristine"[All] OR "Oncovin"[All] OR "Oncovine"[All] OR "Onkocristin"[All] OR "Vincasar"[All] OR "Vincrisul"[All] OR "Vintec"[All] OR "cellcristin"[All] OR "Fauldvincri"[All] Sort by: Publication Date | 33,826 |
| #24 | Search: "Paclitaxel"[MeSH] OR "paclitaxel"[All] OR "Anzatax"[All] OR "NSC-125973"[All] OR "Onxol"[All] OR "Paxene"[All] OR "Praxel"[All] OR "Taxol"[All] OR "NSC125973"[All] OR "NSC125973"[All] OR "Apealea"[All] OR "Paclical"[All] Sort by: Publication Date | 46,287 |
| #25 | Search: "Docetaxel"[MeSH] OR "docetaxel"[All] OR "Docetaxol"[All] OR "NSC 628503"[All] OR "RP 56976"[All] OR "RP-56976"[All] OR "Taxoltere"[All] OR "Taxotere"[All] OR "NSC-628503"[All] OR "NSC628503"[All] OR "RP56976"[All] Sort by: Publication Date | 19,722 |
| #26 | Search: "Cisplatin"[MeSH] OR "cisplatin"[All] OR "cisplatine"[All] OR "Biocisplatinum"[All] OR "Dichlorodiammineplatinum"[All] OR "NSC-119875"[All] OR "NSC 119875"[All] OR "NSC119875"[All] OR "Platidiam"[All] OR "Platino"[All] OR "Platinol"[All] OR "Platinum Diamminodichloride"[All] OR "cis-Diamminedichloroplatinum"[All] OR "cis-Platinum"[All] OR "cis Diamminedichloroplatinum"[All] OR "cis Platinum"[All] Sort by: Publication Date | 87,588 |
| #27 | Search: "carboplatin"[MeSH] OR "carboplatin"[All] OR "carboplatine"[All] OR "Blastocarb"[All] OR "CBDCA"[All] OR "Carboplat"[All] OR "Carbosin"[All] OR "Carbotec"[All] OR "Ercar"[All] OR "JM-8"[All] OR "JM8"[All] OR "JM 8"[All] OR "NSC-241240"[All] OR "NSC241240"[All] OR "NSC 241240"[All] OR "Nealorin"[All] OR "Neocarbo"[All] OR "Paraplatin"[All] OR "Paraplatine"[All] OR "Platinwas"[All] OR "Ribocarbo"[All] Sort by: Publication Date | 20,351 |
| #28 | Search: "Oxaliplatin"[MeSH] OR "oxaliplatin"[All] OR "oxaliplatine"[All] OR "ACT 078"[All] OR "ACT-078"[All] OR "ACT078"[All] OR "Eloxatin"[All] OR "Eloxatine"[All] OR "Dacotin"[All] OR "Elplat"[All] Sort by: Publication Date | 14,899 |
| #29 | Search: #10 OR #11 OR #12 OR #13 OR #14 OR #15 OR #16 OR #17 OR #18 OR #19 OR #20 OR #21 OR #22 OR #23 OR #24 OR #25 OR #26 OR #27 OR #28 Sort by: Publication Date | 323,900 |
| #30 | Search: #9 AND #29 Sort by: Publication Date | 366 |
| #31 | Search: #9 AND #29 Filters: from 1990/1/1 - 3000/12/12 Sort by: Publication Date | 348 |
| #32 | Search: "Drug Interactions"[Mesh] OR "Drug-drug Interactions"[All] OR "Drug Interactions"[All] OR "Drug Interaction"[All] OR "Drug Antagonism"[All] OR "Polypharmacy"[All] OR "Drug Synergism"[All] OR "Drug Synergisms"[All] OR "Drug Potentiation"[All] OR "Drug Potentiations"[All] OR "Drug Augmentation"[All] OR "Drug Augmentations"[All] OR "Drug interaction"[All] OR "Drug interactions"[All] OR "Drug toxicity"[All] OR "Drug toxicities"[All] OR "Drug competition"[All] OR "Drug inhibition"[All] OR "Drug intoxication"[All] Sort by: Publication Date | 205,803 |
| #33 | Search: #31 AND #32 Sort by: Publication Date | 46 |
| #34 | Search: #1 AND #33 Sort by: Publication Date | 5 |
| #35 | Search: #2 AND #33 Sort by: Publication Date | 7 |
| #36 | Search: #3 AND #33 Sort by: Publication Date | 0 |
| #37 | Search: #4 AND #33 Sort by: Publication Date | 0 |
| #38 | Search: #5 AND #33 Sort by: Publication Date | 9 |
| #39 | Search: #6 AND #33 Sort by: Publication Date | 24 |
| #40 | Search: #7 AND #33 Sort by: Publication Date | 7 |
| #41 | Search: #8 AND #33 Sort by: Publication Date | 4 |
| #42 | Search: #10 AND #33 Sort by: Publication Date | 1 |
| #43 | Search: #11 AND #33 Sort by: Publication Date | 0 |
| #44 | Search: #12 AND #33 Sort by: Publication Date | 6 |
| #45 | Search: #13 AND #33 Sort by: Publication Date | 0 |
| #46 | Search: #14 AND #33 Sort by: Publication Date | 3 |
| #47 | Search: #15 AND #33 Sort by: Publication Date | 0 |
| #48 | Search: #16 AND #33 Sort by: Publication Date | 4 |
| #49 | Search: #17 AND #33 Sort by: Publication Date | 0 |
| #50 | Search: #18 AND #33 Sort by: Publication Date | 0 |
| #51 | Search: #19 AND #33 Sort by: Publication Date | 0 |
| #52 | Search: #20 AND #33 Sort by: Publication Date | 0 |
| #53 | Search: #21 AND #33 Sort by: Publication Date | 5 |
| #54 | Search: #22 AND #33 Sort by: Publication Date | 0 |
| #55 | Search: #23 AND #33 Sort by: Publication Date | 7 |
| #56 | Search: #24 AND #33 Sort by: Publication Date | 8 |
| #57 | Search: #25 AND #33 Sort by: Publication Date | 1 |
| #58 | Search: #26 AND #33 Sort by: Publication Date | 15 |
| #59 | Search: #27 AND #33 Sort by: Publication Date | 0 |
| #60 | Search: #28 AND #33 Sort by: Publication Date | 1 |

Searches were undertaken using a combination of medical subject headings (MeSH)
(if available) and/or free text search terms to capture the key concepts of the intervention. Citations were screened online based on their title, title and abstract or full text when available. Results from highlighted rows (green) were selected for data screening and compiling reference lists. Finally, reference lists of all included studies were searched for further relevant studies.

## Data extraction

The following was used as a tool to extract relevant information from outcome evaluations:

1. Journal title and abbreviation

• Not stated

• Details

2. Article/abstract title

• Not stated

• Details

3. Publication date (as well as electronic publication date)

• Not stated

• Details

3. Citation

• Not stated

• Details

4. Author list

• Not stated

• Details

5. Author affiliations

• Not stated

• Details

6. MeSH terms

• Not stated

• Details

7. Target population

• Not stated

• Details

8. Substance (Opioids and anticancer drugs were color-coded)

• Not stated

• Details

9. Keywords

• Not stated

• Details

10. Publication types

• Not stated

• Details

11. PMID/PMCID

• Not stated

• Details

12. DOI

• Not stated

• Details

13. URL

• Not stated

• Details

# References

1. Fallon M, Giusti R, Aielli F, Hoskin P, Rolke R, Sharma M, et al. Management of cancer pain in adult patients: ESMO Clinical Practice Guidelines. Ann Oncol. 2018;29(Suppl 4):iv166-iv91.

2. Chhikara BS, Parang K. Global Cancer Statistics 2022: the trends projection analysis. Chem Biol Lett. 2023;10(1):451-.

3. Sung H, Ferlay J, Siegel RL, Laversanne M, Soerjomataram I, Jemal A, et al. Global Cancer Statistics 2020: GLOBOCAN Estimates of Incidence and Mortality Worldwide for 36 Cancers in 185 Countries. CA Cancer J Clin. 2021;71(3):209-49.
